# Opportunity Cost for Early Treatment of Chagas Disease in Mexico

**DOI:** 10.1371/journal.pntd.0002776

**Published:** 2014-04-17

**Authors:** Janine M. Ramsey, Miguel Elizondo-Cano, Gilberto Sanchez-González, Adriana Peña-Nieves, Alejandro Figueroa-Lara

**Affiliations:** 1 Regional Center for Public Health Research, National Institute for Public Health Research, Tapachula, Chiapas, Mexico; 2 Center for Research in Health Systems, National Institute for Public Health Research, Cuernavaca, Mexico; 3 Salud, Población y Economía AC, Mexico City, Mexico; 4 Epidemiological Research Unit and Health Services, National Medical Center XXI Century, Mexican Social Security Institute, Mexico City, Mexico; Universidad de Buenos Aires, Argentina

## Abstract

**Background:**

Given current neglect for Chagas disease in public health programs in Mexico, future healthcare and economic development policies will need a more robust model to analyze costs and impacts of timely clinical attention of infected populations.

**Methodology/Principal Findings:**

A Markov decision model was constructed to simulate the natural history of a Chagas disease cohort in Mexico and to project the associated short and long-term clinical outcomes and corresponding costs. The lifetime cost for a timely diagnosed and treated Chagas disease patient is US$ 10,160, while the cost for an undiagnosed individual is US$ 11,877. The cost of a diagnosed and treated case increases 24-fold from early acute to indeterminate stage. The major cost component for lifetime cost was working days lost, between 44% and 75%, depending on the program scenario for timely diagnosis and treatment.

**Conclusions/Significance:**

In the long term, it is cheaper to diagnose and treat chagasic patients early, instead of doing nothing. This finding by itself argues for the need to shift current policy, in order to prioritize and attend this neglected disease for the benefit of social and economic development, which implies including treatment drugs in the national formularies. Present results are even more relevant, if one considers that timely diagnosis and treatment can arrest clinical progression and enhance a chronic patient's quality of life.

## Author Summary

Chagas disease is caused by the flagellated protozoan parasite *Trypanosoma cruzi*, vectored in Mexico in both rural and urban areas via one of 18 triatomine bug species. Despite ample morbidity and mortality evidence, however, health policy managers in Mexico have continued to neglect prevention, control and clinical attention for the disease. A computer simulation Markov model was programmed and fed with information from published evidence and an expert panel. The lifetime cost for a timely diagnosed and treated Chagas disease patient is US$ 10,160, while the cost for an undiagnosed individual is US$ 11,877. The cost of a diagnosed and treated case increases 24-fold from early acute to indeterminate stage. The major cost component for lifetime cost was working days lost, between 44% and 75%, depending on the program scenario for timely diagnosis and treatment. Timely medical attention for infected individuals is cheaper than doing nothing, especially if life and labor costs are included. The evidence provided, essential for decision-making, should be used to develop disease-specific prevention, control and patient clinical diagnosis and treatment policies for Chagas disease in Mexico.

## Introduction

Chagas Disease (CD) is caused by the flagellated protozoan parasite *Trypanosoma cruzi* (*T.cruzi*) [1], vectored by triatomine insects known as kissing bugs. The parasite is transmitted most often via the bug's feces, and to a much lesser extent via blood transfusion, congenital or alimentary transmission, and organ transplant or laboratory accident [2], [3].

The disease is endemic in 21 Latin-American countries and the United States, although human migration has expanded at-risk populations for most transmission modes in previously considered non-endemic countries [4]. In Mexico, more than 71,000,000 inhabitants are at direct risk in both rural and urban areas for vector transmission from one of 18 vector species [5], [6]. The current prevalence is not well documented, although most estimates suggest between 0.013%–3.12% of the Mexican population are seropositive [7], [8] and 650,000 chronic cases are currently in some form of clinical care in one of the many health care systems [9].

The first National Seroepidemiology Survey in México, found a 1.6% seroprevalence of antibodies to *T. cruzi* (66,678 samples tested) at the national level. The highest prevalence was found in Chiapas (5.0%), Oaxaca (4.5%) and the south-east region, followed by the central plains of the temperate Huasteca region, which includes the states of Hidalgo (3.2%), San Luis Potosí (2.5%), Veracruz (3.0%) and Tamaulipas (1.6%). However, a limitation of that study was its poor coverage of rural areas, which may have led to a significant underestimate of the current prevalence of the infection and disease [10]. Blood transfusion risk also exists, the review of 64,969 blood donors in 18 states of Mexico, demonstrated a 1.5% seropositivity, with prevalence ranging from 0.2% in Chihuahua up to 2.8% in Hidalgo. A more recent study of blood donations in the Social Security system (IMSS), highlights a similar profile and suggests that in urban populations, 0.4% are seropositive [7]. About 2000 inhabitants each year could be at risk of infection with *T. cruzi* via blood transfusion [11].

Analysis of the economics surrounding a disease can generate information essential for decision-making and evidence-based adoption of specific prevention and control policies. This is particularly useful for health sector authorities in order to generate greater social benefit with a lower cost to the health system [12]. It is also fundamental for creative programming and financing of prevention and control strategies in the face of economic crises and in relation to social and economic development. Direct medical costs to the health system for support therapy for chronic CD cases are remarkable and clinical interventions in the chronic phase raises the costs because it consists of specialized medical care such as palliative and corrective cardiac and digestive surgery [13]. If we consider the indirect costs due to loss of productivity, the burden of CD increases due its impact on individuals in their most productive years [14].

According to the first WHO Report on Neglected Tropical Disease (CD), in Latin America 752,000 working days per year were estimated to be lost due to premature deaths due to CD. The economic cost of CD in terms of lost productivity was estimated at US$ 1.2 billion each year for the seven countries of the Southern Cone. In Brazil, worker absentee affected by CD represents an estimated minimum loss of US$ 5.6 million per year [15].

In Mexico, there is only one published study that estimates the cost of CD treatment; the calculations were based on 13 clinical records at a tertiary level hospital, and hence cost estimates cannot be extrapolated for the entire country or for all healthcare systems [16]. The present study aims to estimate the current costs of treating a chronic CD case detected and treated early vs an undetected case among the salaried population (47% of the Mexican population [17]), and the direct and indirect costs and effects simulated since birth to death using a cohort Markov model.

## Methods

We constructed a Markov decision model based on previous publications [18], [19], to simulate the natural history of a CD cohort and to project the associated short and long-term clinical outcomes. Professional software was used to construct the model (TreeAge Software, Williamstown, Massachusetts).

**Figure 1:**
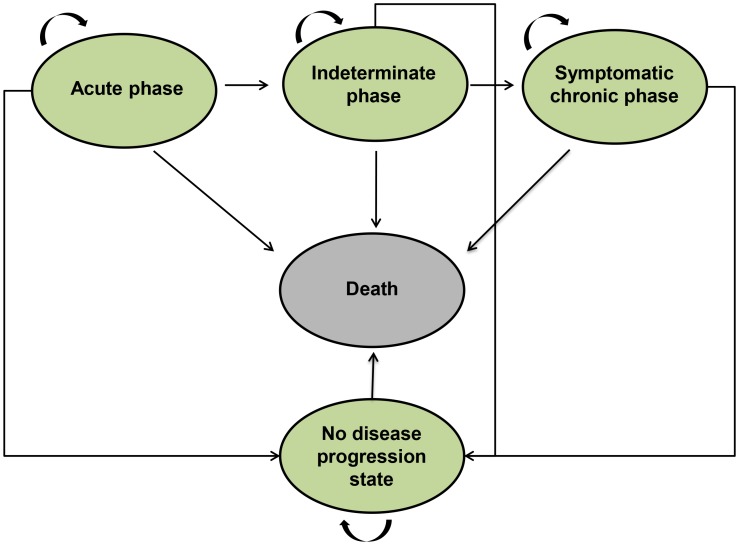
General structure of the Markov model developed. It shows all clinically important events and transition paths from one state to another.

Most recently, several published and ongoing studies have demonstrated that having negative serology after treatment is not a guarantee for remaining seronegative over time [20], [21]. However, given the current lack of evidence validating seroconversion with parasitological clearance and therapeutic cure, we refer in this study to the endpoint for treatment as “no progression”. Figure 1 illustrates the general model structure including the following five Markov states of the disease and an individual's possible transition between states. All clinically important events are modeled as transitions from one state to another using a transition probability [22].

- Acute Phase (AP): The individual is currently infected with *T. cruzi*. Infected individuals remain in this state for a maximum of six months. If there is an effective diagnosis, then the individual could be treated with anti-parasitic drugs (benznidazole) and moves to the no progression state as a function of age. A positive serology implies remaining in the disease state (acute, indeterminate or chronic). When there is no diagnosis or the treatment fails, the person faces the probability of developing myocarditis or/and meningoencephalitis (minor children) that could lead to death. When the AP terminates, the person enters into the indeterminate asymptomatic phase. The initial age of the cohort is 10 years old.
- Indeterminate Asymptomatic Phase (IAP): this is subsequent to the acute phase. Those who become infected can remain in this phase for at least 10 years before transitioning to a chronic form of CD, or remain in this phase for life. If indeterminate patients are diagnosed and treated (benznidazole), and develop negative serology, they move to no progression state. However, if they are positive serologically after treatment, they remain in a disease state.
- Symptomatic chronic phase (SCP): individuals face the probability of developing a cardiomyopathy alone, a megaesophagus and/or a megacolon, or either symptoms together with the first. The probabilities of occurrence of these symptoms are time rate variables. In the model we assume that only the cardiomyopathy can lead to dead, and the patient can only undergo surgery for megaesophagus, megacolon or pacemaker placement.
- No progression state: patients who have negative serology. These patients may come from the acute, indeterminate or chronic phase.
- Death: death occurs as a result of either CD phases (acute or chronic) or other causes unrelated to *T. cruzi* infection.

Each cycle length is one month in the acute phase and then it switches to a year for the rest of disease phases. All Markov states are mutually exclusive. Transition can occur from one state to another during each cycle (Figure 1). Patients are absorbed into the death state, where they remain, not being allowed to transition to another state. The simulation is run until the entire cohort dies.

We compared three detection and treatment scenarios: (1) 100% individuals are detected and treated early (who are diagnosed and treated during the acute phase of the disease), (2) 100% of individuals are detected but only 80% are given treatment (the latter scenario was developed to include patient refusal and/or consideration for those patients not clinically capable of treatment for concomitant health reasons), and (3) no one is diagnosed or treated. The comparative performance was assessed by summing direct costs for medical treatment and indirect costs. We used a modified social perspective, in that costs of patients' time and travel were not included. Future costs were discounted at 5% per year. The discount rate is a financial adjustment which is applied to determine the present value of a future payment and differs from the rate of interest, in that it applies to the original amount for the increase.

A second order Monte Carlo simulation was used in which disease progression in an individual is characterized as a sequence of transitions between health states. One million patients were simulated, one at a time, in order to provide stable estimates of long-term outcomes for each strategy. All the parameters used to feed the model were introduced as statistical distributions: costs inputs are set as gamma distributions and probabilities of transition are beta distributed. Because in a second order calculation all these distributions are sampled, no sensitivity analysis is necessary.

Baseline estimates for selected variables were developed from information provided from published studies and an expert panel (Table 1 to Table 3). Given the lack of information regarding medical care consumption by CD patients in Mexico (direct costs), we consulted an expert panel of four experts. All of them with at least five years of experience in CD in Mexico. The first expert is a physician with experience in CD patient care in the state of Morelos, at the moment we consulted him he was vector control manager, which includes CD disease. The second expert is a health researcher and physician with experience in CD patient care in the state of Jalisco. The third expert is a health researcher and epidemiologist in the state of Jalisco, her main research line is CD. The fourth expert is a physician with experience in CD patient care in the state of Veracruz. All participants were sent instructions with a set of three (one per CD phase) forms to complete. Medical care and procedures for all phases were obtained by a panel of three clinical experts (none of the experts are also authors of this manuscript).

**Table 1:** Model parameters: Baseline estimates for selected variables, acute phase.

Disease phase	Type of parameter	Parameter description	Value	Source of data
Acute	Clinical	Initial average age	10 year old	Expert panel
		Death for other causes	Mexican death tables	[35]
		Develop myocarditis and meningoencephalitis	5%	[36]
		Negative seroconversion (Benznidazole for CD treatment)	53%–70%	[37], [38]
		Death due meningoencephalitis or myocarditis	<5–10%	[39]
		Stage Length	6 months	Expert panel
	Cost* (2012 US)	Medical counseling	$17	[24] and expert panel
		Hospitalization	$6.5	[24] and expert panel
		Laboratory test	$12.4	[24] and expert panel
		Imageneology	$2.6	[24] and expert panel
		Drug treatment**	$61.2	[24] and expert panel
		Annual screening	$5.7	[40] and expert panel

Notes: CD = Chagas disease;

*Yearly cost; US = United States Dollar.

**Only first year.

**Table 2:** Model parameters: Baseline estimates for selected variables, indeterminate phase.

Disease phase	Type of parameter	Parameter description	Value	Source of data
Indeterminate	Clinical	Negative seroconversion (Benznidazole for CD treatment)	19.1%	[41]
		Stage Length	15–30 years	Expert panel
	Cost* (2012 US)	Medical counseling	$16.70	[24] and expert panel
		Hospitalization	$0	[24] and expert panel
		Laboratory test	$28.6	[24] and expert panel
		Imageneology	$4.2	[24] and expert panel
		Drug treatment**	$99.8	[24] and expert panel
		Working days lost due to CD per year	5	[29]
		Value of working days lost	$94.9	[29] and expert panel

Notes: CD = Chagas disease;

*Yearly cost; US = United States Dollar.

**Only first year.

**Table 3:** Model parameters: Baseline estimates for selected variables, chronic phase.

Disease phase	Type of parameter	Parameter description	Value			Source of data
Chronic	Clinical					
		Develop severe heart disorders	25% to 30%			[42]
		Cardiopathy no progression due to drug treatment	88.9%			[43]
		Develop digestive symptoms (megaesophagus or megacolon)	5.5%			[44]
		Develop megaesophagus	10%–20% of infected patients			[45]
		Death due cardiopathy	70%			[46]
		Surgery due to megaesophagus	2%			Expert panel
		Stage Length	10–20 years			Expert panel
	Cost* (2012 US)		Normal	Cardiopathy	Megaesophagus/Megacolon	
		Medical counseling	$68.1	$481.2	$296.6	[24] and expert panel
		Hospitalization	$21.4	$151.2	$93.2	[24] and expert panel
		Laboratory test	$36	$254.4	$156.8	[24] and expert panel
		Imageneology	$25.4	$179.9	$110.9	[24] and expert panel
		Drug treatment**	$162.8	$162.8	$162.8	[24] and expert panel
		Megaeshophagus or Megacolon surgery***	$12,219.1	$12,219.1	$12,219.1	[24] and expert panel
		Placement of pacemaker***	$18,123	$18,123	$18,123	[47]
		Working days lost due to CD per year	12	14	15	[29]
		Value of working days lost	$232.5	$265.7	$284.7	[29] and expert panel

Notes: CD = Chagas disease;

*Yearly cost (2012 US);

US = United States Dollar;

**Only first year;

***Per event.

Costs include both direct and indirect costs. Direct medical costs for CD include those for hospitalization, outpatient consultations, laboratory tests, annual screening, clinical procedures, and medications. Undiagnosed patients have the national average medical care consumption. If a patient develops meningoencephalitis, myocarditis and/or megasyndromes (megaeshophagus, megacolon), medical attention is calculated for these specific symptoms. Average medical care consumption was estimated using the National Survey of Health and Nutrition 2012 (ENSANUT for its acronym in Spanish) [23]. Costs of medical care were built using pricing from the Mexican Social Security Institute (IMSS) [24]. Total costs were obtained by multiplying the quantity of services consumed by the unit costs. The indirect costs, were calculated considering the IMSS average daily wage (US$ 18.9) and the average working days lost due to illness [25]. The initial age of the cohort is 10 year old and we assume that children do not have remunerated work, so we did not consider working days lost due CD in the acute phase.

All costs are expressed as 2012 value of the US dollar or after foreign currency conversion, using average annual exchange rates provided from the International Monetary Fund [26]. Once converted into US dollar, costs were adjusted for inflation using the US Consumer Price Index [27]. The average cost per patient for each phase of the disease was calculated considering the entire cohort of patients, regardless of the phase of the disease that the patient had reached. Subsequently, we calculated the average cost per patient for each phase of the disease, and considered only patients who achieved the phase.

## Results

The average cost per patient, considering the entire cohort of patients, for each of the scenarios is included in Table 4. In the acute phase, the greatest cost per patient occurs for those in the 80% diagnosed and treated scenario (US$ 234), with little difference compared to the scenario where 100% of patients are diagnosed and treated (US$ 232). The category costs for timely diagnosis and treatment in the former group is US$ 31 for medical counseling, US$ 83 for hospitalization, US$ 66 for laboratory tests (blood chemistry, urine test, complete blood count, urea, creatinine, indirect haemagglutination test, etc), US$3 for radiology and imaging, and US$ 51 for drugs (benznidazole for CD treatment and other drugs).

**Table 4:** Average cost and confidence interval (95%) per patient per lifetime according to disease phase, by cost category.

		Category of cost (2012 US)						
Disease phase	Strategies of Dx and Tx	Medical counseling	Hospitalization	Laboratory test and diagnosis	Imaging	Drugs	Value of working days lost	Total
Acute	Dx and Tx of 100%	36 (32–39)	79 (71–87)	67 (60–74)	4 (4–4)	47 (42–51)	0 (0–0)	232(209–267)
	Dx and Tx of 80%	31 (28–34)	83 (74–91)	66 (60–73)	3 (3–4)	51 (46–56)	0 (0–0)	234 (211–269)
	Undiagnosed	6 (6–7)	50 (45–55)	30 (27–33)	0 (0–0)	34 (31–37)	0 (0–0)	120 (108–138)
Indeterminate	Dx and Tx of 100%	289 (260–318)	2,094 (1,884–2,303)	1,276 (1,884–2,303)	3 (1,884–2,303)	1,423 (1,884–2,303)	557 (501–641)	5,641 (5,077–6,488)
	Dx and Tx of 80%	333 (300–366)	2,414 (2,173–2,656)	1,471 (1,324–1,618)	3 (3–4)	1,641 (1,477–1,805)	642 (578–739)	6,505 (5,854–7,481)
	Undiagnosed	109 (98–120)	840 (756–924)	508 (457–559)	0 (0–0)	573 (515–630)	1,279 (1,151–1,471)	3,309 (2,978–3,805)
Chronic	Dx and Tx of 100%	23 (21–26)	176 (159–194)	81 (73–89)	1 (1–1)	86 (78–95)	3,919 (3,527–4,506)	4,287 (3,858–4,930)
	Dx and Tx of 80%	24 (21–26)	180 (162–198)	78 (70–86)	2 (1–2)	82 (74–91)	4,453 (4,008–5,121)	4,819 (4,337–5,542)
	Undiagnosed	36 (33–40)	559 (503–615)	125 (112–137)	4 (3–4)	138 (125–152)	7,586 (6,828–8,724)	8,449 (7,604–9,716)

Notes: US = United States Dollar; Dx = Diagnosis; Tx = Treatment.

In the indeterminate phase of the disease, the timely diagnosis and treatment in 80% of patients generates an average US$ 6,505 cost per patient, while the timely diagnosis and treatment of 100% of patients generates an average of US$ 5,641. The average cost per patient not receiving diagnosis or treatment is US$ 3,309, since they do not have CD specific medical care.

The most expensive chronic phase scenario occurs due to undiagnosed patients, US$ 8,449, which includes working days lost. The cost per patient for the diagnosis and treatment of 80% of patients is US$ 4,819 and the least expensive scenario is where all patients are diagnosed and treated (US$ 4,287).

If all costs per patient are compared among the three program scenarios, early diagnosis and treatment of 100% of CD cases results in a lifetime costs US$ 10,160 (US$ 232+US$ 5,641+US$ 4,287). The lifetime cost per patient if only 80% are diagnosed and treated is US$ 11,558 (US$ 234+US$ 6,505+US$ 4,819), and if no patient is diagnosed or treated, the cost is US$ 11,877 (US$ 120+US$ 3,309+US$ 8,449). The major cost components for the 100% and 80% scenarios are working days lost (44%), followed by hospitalization (23%), drugs (15%), laboratory (14%), and medical counseling (3%). However, for the undiagnosed scenario, the major cost component of working days lost rises to 75%, followed by hospitalization (12%), drugs (6%), laboratory test and diagnosis (6%), and medical counseling (1%).

The cost of a diagnosed and treated case increases 24-fold from early acute to indeterminate stage (100% scenario). The cost per patient in the indeterminate stage is 1.32 fold, more than the cost in the chronic stage (100% scenario). The costs for the undiagnosed patient scenario are systematically lower than either of the 100% treated for acute and indeterminate phases (1.93 and 1.70 times, respectively), due to treatment-specific costs. However, in the chronic phase, the undiagnosed patient scenario incurs most costs, being 1.97 times greater than in the 100% treatment scenario.

The phase specific cost per patient per year is summarized in Table 5. While the results for the acute phase are the same as shown in the previous table, in the indeterminate phase, the average cost per patient is greater for the 80% diagnosed and treated early alternative (US$ 12,772). In the chronic phase, the average cost is greater for the alternative where 100% of patients are diagnosed and treated, with a total of US$ 24,588.

**Table 5:** Cost per patient per year and confidence interval (95%), considering only patients in each phase.

Disease phase	Proportion of patients diagnosed and treated	Cost (2012 US)
Acute	100%	232 (209–255)
	80%	234 (211–257)
	0%	120 (108–132)
Indeterminate	100%	12,765 (11,488–14,041)
	80%	12,772 (11,495–14,050)
	0%	3,502 (3,152–3,852)
Chronic	100%	24,588 (22,129–27,047)
	80%	23,929 (21,536–26,321)
	0%	16,630 (14,967–18,293)

Notes: US = United States Dollar.

## Discussion

The costs of a chronic CD case detected and treated vs an undetected case has been analyzed herein from a modified social perspective which allows us to take into account the value of working days lost. We calculated two different types of costs: costs per patient per lifetime according to disease stage (or cohort cost, discounted at a rate of 5%) and costs per patient per year. The lowest lifetime cost is estimated from the 100% early diagnosis and treatment scenario, due to the fact that in this scenario, less of the cohort reaches the expensive chronic phase of the illness. It is important to stress that the parallel costs between the 100% and the 80% treated scenarios is because both populations are diagnosed and have similar medical management.

The phase specific costs increase accordingly with each progressive phase for all scenarios, although the cost estimated for the undiagnosed category is less in chronic phase than that for either 80% or 100% scenarios (US$ 16,630 vs. US$ 23,929 or US$ 24,588, respectively). Although surprising, this result is considered real, since there is high mortality in the undiagnosed patient group, and since costs are calculated for a complete cohort, they will be proportionally reduced due to patients lost to the cohort (and hence reduced average patient cost).

Vallejo et al. [16] reported the cost of medical treatment of 13 clinical cases for CD in a specialized hospital setting (third level) in Mexico. The annual cost for medical care for patients in this outpatient setting was estimated between US$ 4,463 and US$ 9,601, and annual costs for patients admitted via an emergency care unit was between US$ 6,700 and US$ 11,838. If we assume that these patients were in the chronic phase of the disease, the costs we calculated herein are similar to their higher costs. Contrary to our findings, Vallejo et al. conclude that highest cost components were radiology and imaging (63%) and hospitalization (26%), while the component with least contribution to cost was drug treatment (3%). The limitations of the previous study are the few patients used to estimate costs, the costs of surgeries (pacemaker placement were not considered), and the bias for disease phase, since in order to be attended in a specialized hospital, patients must have economic capacity (to afford out-of-pocket expenses to go to Mexico City for varying periods), and must be in the chronic phase with cardiomyopathy.

The present study uses second and third level social security (IMSS) costs as an alternative and complementary perspective for opportunity costs of diagnosis and treatment, since the IMSS system currently covers 47% of the Mexican population, and in the poorest states (Chiapas, Campeche, Yucatan), the IMSS Oportunidades subsystem still covers at least half of the rural population [17]. Future studies should also focus on the Seguro Popular and the primary healthcare system, Secretariate's second level hospitals and costs generated in these systems. Until there is a more robust estimate of patient population seroprevalence in all Mexican healthcare groups and regions, the costs calculated herein may be considered pertinent particularly for populations with fixed incomes.

In other countries such as Colombia, chronic CD cost has been calculated from the payer's perspective (review of 63 clinical records) [28]. Castillo-Riquelme et al. concluded that cost per patient per year for clinical management at the basic care level was US$ 46 to US$ 51, the cost in the intermediate level of care was US$ 188 to US$ 259, and in a specialized setting the cost per patient per year was US$ 3,652 to US$ 7,981. If we compare the costs per patient per year of the acute phase of the 100% and 80% scenarios they are similar to those reported by Castillo-Riquelme et al. for their intermediate level of care. The costs for chronic phase reported in the present study is between 3 and 6.7 times greater than costs reported by Castillo-Riquelme et al. The difference may be due to the methodological perspective which in the latter was based on the provider and the present study based a modified social perspective. The value for worked days lost was 91.4% of the total costs in the 100% scenario in the chronic phase in the present study.

Castillo-Riquelme et al estimated for the intermediate level of care, hospitalization contributes between 25% and 49%, drugs contribute 31% to 42% and for specialized care, the surgical procedures were the largest cost component, contributing between 41% and 55%, while the second largest was drugs (10% and 24%). The distribution of the total costs reported by the present study are similar to those reported by Castillo-Riquelme et al. except for the fact that the major cost component in the present study was the value of working days lost (44%), followed by hospitalization (23%) and drugs (15%).

Basombrio et al. [29] reported the direct and indirect (value of working days lost) cost of CD in Argentina and concluded that acute phase costs were US$ 591 per patient per year, of which 34% corresponded to medical counseling and 27% to labor loss. The indeterminate phase cost was US$ 174, of which 30% corresponded to labor loss and 28% to laboratory tests, similar to that reported herein. Chronic phase costs were between US$603 and US$ 736, of which 27% to 37% corresponded to medical counseling and 21% to 23% to labor loss, a significant difference with the proportion estimated with the present study. Contrary to data reported by Castillo-Riquelme, and similar to the present study, the contribution for surgery was between 1% and 8% [28], [29]. Hence, labor loss costs in Argentina represent approximately 25% of total costs across all disease stages, while in the present findings, labor loss in indeterminate is higher than this, but becomes the largest cost component in the chronic stage. Schenone reported that average annual patient costs for chronic chagasic cardiopathy in Chile is between US$ 439 and US$ 584, while we estimated a cost between US$ 16,630 and US$ 24,588 [30]. The previous study did not consider labor loss, which may in part account for these differences.

Based on information gathered from the literature review and expert panel, Akhavan estimated in Brazil that the lifetime medical care cost of a chagasic patient in the indeterminate phase is US$ 1,140, the cost for a patient with digestive complication was between US$ 4,510 and US$ 9,890, while the cost for a patient with cardiac complications was US$ 4,075 to US $55,159. Unfortunately, that study does not provide the distribution of the cost components [31]. The lifetime cost per patient in the indeterminate phase estimated by the present study ranges from US$ 6,488 to US$ 7,481, which is 5.7 to 6.6 times greater than the costs estimated by Akhavan. In addition, the costs estimated by Akhavan do not include the value of working days lost. The lower limit cost estimated by Akhavan for the digestive and cardiac complications, which both occur in the chronic phase, are similar to the cost estimated by the present study. The upper limit costs from Akhaven vary between 1.8 to 11.2 times greater than the costs estimated in the present study. This difference can be explained due to the fact that costs in the previous study were calculated based on medical care consumption, and in the present study a cohort was used.

Using a methodological approach similar to the present study, Lee et al. [32] estimated the global economic cost of CD from a societal perspective even though they do not report cost specifically for Mexico and they do not consider the only other CD cost study from Mexico for their analysis [16]. For Latin America, the annual health-care cost per patient was US$ 383 (range: US$ 207–US$ 636), annual cost per patient due to productivity losses was US$ 3,676 (range: US$ 3,362–US$ 3,798), that is to say that productivity losses were estimated at 9.6 times greater than the medical care cost (direct cost). The lifetime cost per patient for an individual with CD was estimated at US$ 2,600 (range: US$ 1,966–US$ 3,034). The lifetime cost estimated in the present study is 3.9 times greater than that calculated by these authors.

Present data suggest that in the long term, it is cheaper to appropriately diagnose and treat chagasic patients instead of doing nothing. This finding by itself should motivate public policy to attend and appropriately manage exposed and potentially infected populations and establish public health interventions for this disease in Mexico, which has been neglected by health authorities [33], [34]. This finding is even more convincing if one considers that appropriate anti-parasitic treatment can arrest further progression of disease and enhance, in the case of chronic cases, the patient's quality of life. The short and long term labor context and impact of the disease should be more carefully analyzed and considered by labor management and economic strategists, as in the case of other neglected tropical diseases, especially when public policy prioritized evidence-based social en economic development.

One of the important limitations of the present study, a reflection regarding the almost complete absence of this disease in the medical care and public health community in Mexico, was the reduced pool of clinical experts in order to construct more robust clinical care models. Once Mexico publishes a clinical guideline for CD, and if there is a decision to revert the neglect for the disease at all levels of health care and preventive public health programs, more complete analysis can consider the heterogeneity and real costs for all sectors of the Mexican population. Chagas disease is a neglected tropical disease, internationally, and particularly in Mexican public health policy. The implications of continued abandonment to prevent and attend exposed population should be evaluated from both individual and collective perspectives and from all sectors, so that its impact at all levels of the Mexican economy can be considered for evidence-based policy decisions.

## References

[1] Diaz J (2008) Recognizing and reducing the risks of Chagas disease (*American trypanosomiasis*) in travelers. J Travel Med 15: 184–195. 18494696 10.1111/j.1708-8305.2008.00205.x

[2] Prata A (2001) Clinical and epidemiological aspects of Chagas disease. Lancet Infect Dis 1: 92–100. 11871482 10.1016/S1473-3099(01)00065-2

[3] World Health Organization (2012) Chagas disease (*American trypanosomiasis*) fact sheet N° 340, august 2012. Available:http://www.who.int/mediacentre/factsheets/fs340/en/index.html. Accessed 10 October 2012.

[4] Coura JR, Viñas PA (2010) Chagas disease: a new worldwide challenge. Nature 465: s6–s7. 20571554 10.1038/nature09221

[5] Ramsey JM, Cruz A, Salgado L, Espinosa L, Ordoñez R, et al (2003) Efficacy of pyrethroid insecticides against domestic and peridomestic populations of *Triatoma pallidipennis* and *Triatoma barberi* (*Reduviidae:Triatominae*) vectors of Chagas' disease in Mexico. J Med Entomol 40: 912–920. 14765670 10.1603/0022-2585-40.6.912

[6] Ibarra-Cerdeña CN, Sánchez-Cordero V, Townsend Peterson A, Ramsey JM (2009) Ecology of North American Triatominae. Acta Trop 110: 178–186. 19084490 10.1016/j.actatropica.2008.11.012

[7] Novelo-Garza BA, Benítez -Arvizu G, Penña-Benítez A, Galván-Cervantes J, Morales-Rojas A (2010) Detección de *Trypanosoma cruzi* en donadores de sangre. Rev Med Inst Mex Seguro Soc 48: 139–144. 20929616

[8] Segura E, Escobar-Mesa A (2005) Epidemiología de la enfermedad de Chagas en el estado de Veracruz. Salud Publica Mex 47: 201–208. 16104462 10.1590/s0036-36342005000300003

[9] López T, Panzera F, Tun E, Ferrandis I, Ramsey J (2009) Contribuciones de la genética y la proteómica al estudio de la enfermedad de Chagas. Salud Publica Mex 51: S410–S423. 20464215 10.1590/s0036-36342009000900007

[10] Velasco O, Valdespino J, Tapia R, Salvatierra B, Guzmán C, et al (1992) Seroepidemiología de la Enfermedad de Chagas en México. Salud Publica Mex 34: 186–196. 1631732

[11] Guzmán C, García L, Florián J, Guerrero S, Torres M, et al (1998) Riesgo de transmisión de *Trypanosoma cruzi* por transfusión de sangre en México. Rev Panam Salud Publica 4: 94–99. 9810428 10.1590/s1020-49891998000800004

[12] Drummond M, O'brien B, Stooddart G, Torrance G (2001) Métodos para la evaluación económica de los programas de asistencia sanitaria. Madrid: Ediciones Díaz de Santos.367 p.

[13] Castillo M (2007) Dimensión de costos de la Enfermedad de Chagas. In: Rosas F, Vanegas D, Cabrales M. Enfermedad de Chagas. Bogotá, DC: Sociedad Colombiana de cardiología y cirugía cardiovascular. pp. 205–215.

[14] Rosas F, Guhl F, Luquetti A (2007) Tratamiento etiológico de la Enfermedad de Chagas. In: Rosas F, Vanegas D, Cabrales M. Enfermedad de Chagas. Bogotá, DC: Sociedad Colombiana de cardiología y cirugía cardiovascular. pp. 139–143.

[15] World Health Organization (2010) Working to overcome the global impact of neglected tropical diseases. First WHO report on neglected tropical disease. France: World Health Organization.172p.

[16] Vallejo M, Montenegro P, Reyes PA (2002) How much does the medical treatment of chronic Chagas cardiopathy cost? Direct costs in a cardiology hospital. Arch Cardiol Mex 72: 129–37. 12148332

[17] Pérez-Cuevas R, Doubova SV, Suarez-Ortega M, Law M, Pande AH, et al (2012) Evaluating quality of care for patients with type 2 diabetes using electronic health record information in Mexico. BMC Med Inform Decis Mak 12: 50. 22672471 10.1186/1472-6947-12-50 PMC3437217

[18] Lee BY, Bacon KM, Connor DL, Willig AM, Bailey RR (2010) The potential economic value of a *Trypanosoma cruzi* (Chagas disease) vaccine in Latin America. PLoS Negl Trop Dis 4: e916. 21179503 10.1371/journal.pntd.0000916 PMC3001903

[19] Wilson LS, Strosberg AM, Barrio K (2005) Cost-effectiveness of Chagas disease interventions in latin america and the Caribbean: Markov models. Am J Trop Med Hyg 73: 901–910. 16282301

[20] Machado-de-Assis GF, Diniz GA, Montoya RA, Dias JC, Coura JR, et al (2013) A serological, parasitological and clinical evaluation of untreated Chagas disease patients and those treated with benznidazole before and thirteen years after intervention. Mem Inst Oswaldo Cruz 108: 873–880. 24037109 10.1590/0074-0276130122 PMC3970640

[21] Rodriques-Coura J, de Castro SL (2002) A critical review on Chagas disease chemotherapy. Mem Inst Oswaldo Cruz 97: 3–24. 11992141 10.1590/s0074-02762002000100001

[22] Sonnenberg FA, Beck JR (1993) Markov models in medical decision making: a practical guide. Med Decis Making 13: 322–338. 8246705 10.1177/0272989X9301300409

[23] Secretaría de Salud de México (2012) Encuesta Nacional de Salud y Nutrición 2012. Available: http://ensanut.insp.mx/basesdoctos.php#.Umb8lvkz2QM. Accesed 8 August 2013.

[24] Secretaría de Gobernación (2011) Diario Oficial de la Federación, martes 14 de junio 2011. Available: http://dof.gob.mx/nota_detalle.php?codigo=5296312&fecha=18/04/2013. Accesed 10 January 2013.

[25] Instituto Mexicano del Seguro Social (2013) Salario promedio diario. Available: http://www.stps.gob.mx/bp/secciones/conoce/areas_atencion/areas_atencion/web/menu_infsector.html. Accessed 10 January 2013.

[26] International Monetary Fund (2009) International exchange rates. Washington, DC: IMF.

[27] US Bureau of Labor Statistics (2009) Consumer price index: historical dataset. Washington, DC: Bureau of Labor Statistics.

[28] Castillo-Riquelme M, Guhl F, Turriago B, Pinto N, Rosas F, et al (2008) The costs of preventing and treating Chagas disease in Colombia. PLoS Negl Trop Dis 2: e336. 19015725 10.1371/journal.pntd.0000336 PMC2581604

[29] Basombrío MA, Schofield CJ, Rojas CL, del Rey EC (1998) A cost-benefit analysis of Chagas disease control in north-western Argentina. Trans R Soc Trop Med Hyg 92: 137–143. 9764315 10.1016/s0035-9203(98)90720-9

[30] Schenone H (1998) Human infection by *Trypanosoma cruzi* in Chile: epidemiology estimates and costs of care and treatment of the chagasic patient. Bol Chil Parasitol 53: 23–26. 9830720

[31] Akhavan D (1996) Cost-effectiveness analysis of the Chagas disease control programme in Brazil. Project BRA-093/15 with technical Assistance from UN: Brasilia. 37 p.

[32] Lee BY, Bacon KM, Bottazzi ME, Hotez PJ (2013) Global economic burden of Chagas disease: a computational simulation model. Lancet Infect Dis 13: 342–348. 23395248 10.1016/S1473-3099(13)70002-1 PMC3763184

[33] Manne JM, Snively CS, Ramsey JM, Salgado MO, Bärnighausen T, et al (2013) Barriers to Treatment access for Chagas Disease in Mexico. PLos Negl Trop Dis 7: e2488. 24147169 10.1371/journal.pntd.0002488 PMC3798390

[34] Dumonteil E (1999) Update on Chagas's disease in Mexico. Salud Pública Mex 41: 322–327. 10624144 10.1590/s0036-36341999000400010

[35] Consejo Nacional de Población (2012). Tasa de mortalidad general. Available: http://www.conapo.gob.mx/en/CONAPO. Accessed 10 January 2013.

[36] Moreno E, Valerio-Campos I, Goyenaga-Castro P (2007) Miocarditis y miocardiopatía dilatada por *Trypanosoma cruzi*: Reporte de un caso. Parasitol Latinoam 62: 148–153.

[37] Abad-Franch F, Santos WS, Schofield CJ (2010) Research needs for Chagas disease prevention. Acta Trop 115: 44–54. 20227378 10.1016/j.actatropica.2010.03.002

[38] Shikanai-Yasuda MA, Lopes MH, Tolezano JE, Umezawa E, Amato-Neto V, et al (1990) Acute Chagas' disease: transmission routes, clinical aspects and response to specific therapy in diagnosed cases in an urban center. Rev Inst Med Trop Sao Paulo 32: 16–27. 2124370 10.1590/s0036-46651990000100004

[39] Rassi A Jr, Rassi A, Marin-Neto JA (2010) Chagas disease. Lancet 375: 1388–1402. 20399979 10.1016/S0140-6736(10)60061-X

[40] Grupo Jasem (2013) Lista de precios. Available at: http://grupojasem.com/sistema/ca/ListasdePrecios/REPRESA/data/BIORAD%202013.pdf. Accessed 8 February 2013.

[41] Viotti R, Vigliano C, Armenti H, Segura E (1994) Treatment of chronic Chagas' disease with benznidazole: clinical and serologic evolution of patients with long-term follow-up. Am Heart J 127: 151–162. 8273735 10.1016/0002-8703(94)90521-5

[42] Bellini MF, Silistino-Souza R, Varella-Garcia M, de Azeredo-Oliveira MT, Silva AE (2012) Biologic and genetics aspects of Chagas disease at endemic Areas. J Trop Med 357948: 1–11. 10.1155/2012/357948 PMC3317048 22529863

[43] Fabbro DL, Streiger ML, Arias ED, Bizai ML, Del Barco M, et al (2007) Trypanocide treatment among adults with chronic Chagas disease living Santa Fe City (Argentina), over mean follow –up of 21 years: parasitological, serological and clinical evolution. Rev Soc Bras Med Trop 40: 1–10. 10.1590/s0037-86822007000100001 17486245

[44] Teixeira AR, Hecht MM, Guimaro MC, Sousa A, Nitz N (2011) Pathogenesis of Chagas' disease: parasite persistence and autoimmunity. Clin Microbiol Rev 24: 592–630. 21734249 10.1128/CMR.00063-10 PMC3131057

[45] Organización Panamericana de la Salud (2008) Programa regional para el control de la enfermedad de Chagas en América Latina. Iniciativa de bienes públicos regionales. Washington: OPS. 242 p.

[46] Viotti R, Vigliano C, Lococo B, Petti M, Bertocchi G, et al (2005) Clinical predictors of chronic chagasic myocarditis progression. Rev Esp Cardiol 58: 1037–1044. 16185616

[47] Instituto Mexicano del Seguro Social (2011) Grupos Relacionados con el Diagnóstico. México: IMSS. 230 p.

